# The online survey in qualitative research: can AI act as a probing tool?

**DOI:** 10.3389/frma.2025.1519008

**Published:** 2025-09-05

**Authors:** Ryan Thomas Williams, Ewan Ingleby

**Affiliations:** ^1^Teesside University International Business School, TU Online, Teesside University, Middlesbrough, United Kingdom; ^2^School of Social Sciences Humanities and Law, Education Department, Teesside University, Middlesbrough, United Kingdom

**Keywords:** qualitative survey, survey question design, online survey, qualitative research design, artificial intelligence

## Abstract

Surveys are commonly associated with quantitative methods, yet there is growing recognition of their potential to yield qualitative insights into complex social phenomena. However, the effectiveness of open-ended survey questions is often limited by issues such as respondent fatigue and low-quality responses. To address these limitations, researchers are increasingly exploring the use of artificial intelligence (AI) to support dynamic survey design, probing questions, and participant engagement. This article explores the role of qualitative surveys in social science research, by considering their alignment with qualitative paradigms. The content assesses how AI-powered features, such as machine learning and chatbot-driven interfaces, can enhance data collection through adaptive questioning. The article also discusses key challenges related to data quality, participant inclusivity, and ethical considerations. Particular attention is given to the concept of “felt anonymity” in online surveys, which can encourage candid disclosures on sensitive topics and broaden participation across diverse populations. When designed with ethical and methodological care, qualitative surveys can thus serve as powerful tools for accessing underrepresented perspectives. By integrating AI into qualitative survey design, researchers can enhance both the richness and reach of their data. This article argues that AI-powered qualitative surveys, especially those capable of dynamic probing, offer a promising hybrid approach, bridging the scalability of surveys with the responsiveness of interviews, and calls for further empirical study of their ethical and epistemological implications.

## Introduction

Social science researchers frequently adopt surveys and questionnaires as their primary data collection tools. Since the rise of the internet, there has been a transition to online data collection through various survey platforms, such as Qualtrics, Microsoft Forms, and BOS (Williams, 2023). Whilst surveys have traditionally been associated with quantitative research, particularly through Likert and dichotomous question types, mixed-method versions can offer rich qualitative insights to complement and supplement the data. However, the benefits of this are not always realized, as argued by (Braun et al. 2021). This may be because survey instruments are often designed for the collection of continuous or categorical data and do not fully embrace the values and techniques of qualitative inquiry. In other words, surveys frequently overlook the capacity of qualitative data to provide rich, nuanced understandings of social phenomena.

Qualitative surveys typically include open-ended questions that are self-administered by participants, distinguishing them from open-ended interviews, which involve researcher facilitation. However, open-ended survey questions can result in cognitive burdens such as survey-taking fatigue, leading respondents to skip such questions or provide low-quality or irrelevant answers (Ben-Nun, 2008; Chen, 2017). This becomes problematic as it adversely affects the quality and reliability of the data collected, especially when open-ended questions are involved. Several approaches have been proposed to mitigate survey fatigue and encourage participants to provide quality answers to open-ended questions. For example, (Xiao et al. 2020) argue that implementing interactive features, such as response feedback and probing responses, can improve response quality and encourage participant engagement.

Although probing questions have traditionally been associated with semi-structured interviews, technological developments have made it possible for follow-up questions to be presented to the participant based on their response to a previous question (Novick and Gris, 2014). Most survey software now supports branching or skip logic, allowing for customized pathways that improve data credibility and validity. Whilst this strategy has been used in quantitative research, the development of artificial intelligence allows for keywords, length, depth, or answers to generate additional prompts similar to the interview (Xiao et al., 2020). More specifically, machine learning can ask clarification questions where participants can elaborate on their responses to ensure a clear understanding of their answers. For example, if an educator mentions a professional development challenge, the system might prompt them to describe its nature and impact in more detail, thus mimicking the follow-up behavior of a human interviewer.

Similarly, the emergence of chatbots, particularly with their increasingly powerful conversational capabilities, can offer an alternative to static online or AI-powered surveys. Specifically, an artificial intelligence (AI)-powered chatbot can conduct a conversational survey. (Xiao et al. 2020) argue that generative chatbot-powered surveys can pose personalized follow-up messages that improve participant engagement and response quality.

In this article, we challenge the assumption that surveys are inherently unsuitable for qualitative research. We argue that qualitative surveys are indeed compatible with research embedded in broadly qualitative research values and paradigms (Grant and Giddings, 2002; Kidder and Fine, 1987) and that AI-powered qualitative surveys, including those using a chatbot interface, can provide richness and depth when viewed in their entirety, even if individual responses might be brief. This re-conceptualization challenges long-held assumptions about the static nature of surveys and opens a new methodological space for designing scalable, engaging, and ethically attuned qualitative instruments.

## AI in contemporary qualitative research

As artificial intelligence continues to evolve, it is increasingly being incorporated into qualitative research practice. Recent methodological contributions highlight the potential of AI tools to support, and, at times, challenge traditional qualitative processes. These include AI-driven transcription, coding, and even conversational data collection via chatbot interfaces. For example, (Morgan 2023) examine the use of large language models, such as ChatGPT, to generate thematic summaries of qualitative interview transcripts. While promising for efficiency, their findings underscore the need for critical human oversight, particularly regarding contextual accuracy and interpretation. Similarly, (Roberts et al. 2024) explore the application of AI in supporting computer-assisted qualitative data analysis (CAQDAS), suggesting that these tools may enhance data coding but risk depersonalizing the researcher's analytical voice.

The shift toward AI-supported data collection is also gaining momentum. (Zarouali et al. 2024) document the use of chatbot interfaces to conduct structured qualitative interviews. Participants responded positively to the flexibility and perceived anonymity of the bot, but concerns remain regarding empathy and the tailoring of responses. These emerging methods are prompting new ethical considerations and methodological questions for qualitative researchers.

Reflecting this shift, updated methodological texts such as The SAGE Handbook of Qualitative Research (Denzin and Lincoln, 2018) and recent guidance from the UK Academy of Social Sciences (2023) emphasize the importance of balancing AI affordances with core qualitative values such as reflexivity, richness, and situated knowledge. These developments suggest that qualitative researchers are not only adapting to technological innovations however also actively reshaping them to align with the epistemological commitments of their field. When applied thoughtfully and ethically, AI can augment rather than displace the interpretive depth that defines qualitative inquiry.

Despite these benefits, there are notable risks in using chatbots to conduct qualitative surveys. First, turn-by-turn chat formats require participants to invest additional time and effort, which can lead to frustration. Moreover, current literature offers limited evidence that participants are willing to engage in chatbot-based surveys at length or provide high-quality responses (Zarouali et al., 2024). However, user-friendly interfaces and intuitive survey design may help mitigate some of these concerns. (Williams 2023) cautions against overly complex online survey designs, arguing that while graphical enhancements may appear engaging, they can hinder usability—particularly due to increased download times. In other words, the suitability of the interface must be considered in relation to the target audience.

Additionally, as with AI hallucinations, chatbots have inherent limitations in conversational depth, which may contribute to user disappointment and disengagement. Nevertheless, researchers may be well-placed to implement validation and monitoring protocols to detect and address potential errors or biases (Xiao et al., 2020). From a qualitative paradigm perspective, such limitations need not be masked; rather, they can be acknowledged and interpreted reflexively, just as subjectivities are embraced in qualitative interviews.

## What can online qualitative surveys offer?

Qualitative data collection can significantly benefit researchers by facilitating access to participants' perspectives and experiences, which are important for research in the social sciences. The literature indicates that qualitative surveys have been administered to provide insights into the lived experiences particularly in domains such as appearance, sexuality, and health (Davey et al., 2019).

Furthermore, online surveys are a cost-effective and relatively straightforward way to reach broad, geographically scattered populations. This is particularly useful for research with limited funding, time, or reliance on student participation (Braun et al., 2017a). Although this advantage is often associated with quantitative surveys, it applies equally to qualitative approaches. One limitation, however, is that analyzing large qualitative datasets can be time-consuming (Williams, 2024a,b). Software such as NVivo and other forms of computer-assisted qualitative data analysis (CAQDAS) help address this challenge. These tools allow researchers to organize and manage sources in one place, importing and coding different types of data, such as PDFs, Word documents, web pages, audio files, and video (Kelle and Bird, 1995). This demonstrates how qualitative researchers can integrate technology into their work without compromising the values and paradigms that underpin qualitative inquiry.

Such accessibility allows social researchers to collect data from a wider and more varied group of respondents than smaller-scale studies, such as interviews, might achieve. For qualitative researchers, the aim in engaging with diverse participants is often to gain a deeper understanding of the subject matter rather than to produce a statistically representative sample. Nevertheless, such research can still support broader inferences, which is often a consideration for both quantitative and qualitative designs (Terry and Braun, 2016; Terry et al., 2018).

Including diverse perspectives is central to the quality and validity of research findings and also influences how knowledge can be applied in practice. (Suzuki et al. 2007, p. 295) encapsulates the analogy, “the pond you fish in determines the fish you catch” to describe this phenomenon. Online qualitative surveys can extend researchers' reach beyond typical, more accessible participant groups, enriching studies with a broader spectrum of voices (Terry and Braun, 2017). They may also benefit those with mobility or time constraints, for instance. While virtual interviews on platforms such as Microsoft Teams have addressed some traditional accessibility issues, surveys can still offer a more flexible alternative.

Although qualitative surveys offer significant advantages for inclusivity and participant engagement, they also present a notable drawback due to the literacy requirement, potentially excluding individuals with limited reading and writing skills (Zarouali et al., 2024). Encouraging participants by emphasizing that perfect spelling or grammar is unnecessary can help mitigate this issue (Williams, 2023). Additionally, scaffolding techniques that include clear instruction to help participants navigate the questions, clarification of terms and acronyms, examples and prompts, and progress bar indicators can further support participants and provide guidance to help them engage more effectively with the survey questions and provide meaningful responses (Song and Kim, 2021).

It is also essential to acknowledge the digital literacy gap when administering qualitative surveys over interviews. (Van Deursen and Van Dijk 2019, p. 75) argue that “the digital divide is not just about access to the internet, but also about the capacity to use it effectively for social and economic benefit”.

In other words, qualitative surveys may exclude the most disadvantaged and vulnerable populations from participating in online research (Van Deursen and Van Dijk, 2019). This is particularly important in research methodologies that are justified by arguing that qualitative surveys increase inclusivity by default. Researchers must consider these factors during the survey design process to minimize exclusion and bias.

Another common critique of qualitative surveys is a perceived lack of depth compared to other qualitative strategies, such as interviews. However, (Braun and Clarke 2006) argue that this viewpoint may stem from a misconceived notion about what qualitative surveys cannot provide, coupled with an overestimation of the capabilities of interviews. In reality, qualitative surveys can yield similarly rich, intricate, and profound data. Indeed, whilst interviews focusing on personal narratives on sensitive issues may yield informative and detailed data, there may be no meaningful reflections on their experiences to couple with the detailed accounts. This is due to the complexity of emotional intensity that is associated with interviews. Emotional complexity can sometimes be more densely expressed in survey responses (Davey, 2019). (Braun and Clarke 2006) argue that shifting the perspective toward emphasizing the overall richness of the dataset is better than focusing solely on the amount of detail provided by individual data points. In other words, once researchers acknowledge the limitations of interviews, they may be more open to embracing a qualitative survey.

## Encouraging disclosure and participation in sensitive topics

Online qualitative surveys have the potential to give voice to participants who might otherwise abstain from face-to-face research due to the sensitive nature of the topic or project. The data quality obtained from participants unwilling to engage in methods such as interviews should not be underestimated (Davey et al., 2019, p. 12). Interestingly, (Wiederman 1997) found that questionnaire studies were more appealing to potential participants than face-to-face interviews or laboratory studies. (Braun et al. 2017a,b); (Braun and Clarke 2013) argue that qualitative surveys are similarly well suited to sensitive research as they offer the participants high anonymity (Terry and Braun, 2017).

Researchers often face the dilemma of wanting to guarantee anonymity to encourage participation during data collection. Nevertheless, online surveys can create a sense of “felt anonymity” from the participant's perspective. As most research now includes withdrawal dates for participants, the researcher must be able to trace the participants back to the data in case they wish to withdraw. It is worth noting that, in practice, online services are similarly not completely anonymous, as software can capture IP addresses (Williams, 2023). However, the online survey can nonetheless “feel” completely anonymous from the participant's perspective. Moreover, felt anonymity refers to the perception of being unidentifiable, even when anonymity is not technically guaranteed.

Felt anonymity can influence participants' willingness to disclose personal information, share sensitive experiences, or express candid opinions. Even if participants know that their identity may not be completely hidden, the perception of anonymity can still make them feel more comfortable and less inhibited in their responses. In other words, the researcher cannot see them and does not know their name, which can facilitate participation and disclosure in sensitive research (Braun et al., 2021; Terry and Braun, 2017).

(Braun et al. 2021) compared video interviews with survey responses to support this notion and found that participants could provide more explicit details about their thoughts and feelings during the surveys. This supports the argument that felt anonymity can facilitate intimate disclosures, even when the survey precedes the interview.

Whilst felt anonymity can help create a safe space for participants to share their perspectives, enhancing the quality and depth of qualitative data collected, the researcher must consider the ethical implications. Participants should be clearly informed about the extent of anonymity provided and any associated risks. Researchers should communicate how participant data will be handled, stored, and used to ensure participants can make informed decisions about their involvement. Similarly, researchers must take the same precautions to protect the confidentiality of participant data, even if participants perceive themselves as anonymous. This includes securing data against unauthorized access and ensuring participants cannot be personally identified from their responses, and obtaining explicit consent for any data sharing or publication.

Social comfort is an element that may facilitate disclosure, participation, and data quality with qualitative surveys. In other words, only some people feel comfortable in face-to-face interactions. Online qualitative surveys can serve populations who experience high levels of anxiety around social interaction (e.g., women with OCD, as highlighted by Braun et al., 2021). Likewise, online surveys can work well for topics where participants might not want to be visible to or feel scrutinized by the researcher (e.g., people with a visible difference) or when face-to-face data collection might be “triggering”.

Finally, surveys require fewer interpersonal skills from researchers than interviews, such as fostering a rapport with the participants, meaning they avoid some ethical concerns around inexperienced researchers interacting with (potentially vulnerable) participants and asking invasive or ‘triggering' questions (Braun and Clarke, 2013; Braun et al., 2017a; Terry and Braun, 2017).

We argue that surveys still require careful consideration and ethical oversight, particularly in the design phase, to ensure that questions are formulated sensitively and do not inadvertently cause distress or trigger negative emotions among respondents. Additionally, the absence of direct interaction in surveys does not guarantee ethical integrity; rather, it shifts the responsibility to the questionnaire design and distribution process.

Furthermore, scholars argue that interviews allow direct interaction between researchers and participants, and this provides an opportunity for real-time clarification, empathy, and ethical oversight during the data collection process (Cohen et al., 2017). If probing questions are generated through artificial intelligence, there is a risk they may prioritize logical flow over emotional nuance or empathy.

## AI as a probing tool

Like qualitative interviews, the primary aim of surveys is to elicit rich information from a target audience, often best achieved through some form of dialogue (Cohen et al., 2017). Artificial intelligence, particularly generative chatbots, has been proposed as a solution to some limitations of using surveys for qualitative data collection and, more importantly, to generate conversation. These technologies represent a methodological shift toward bridging the rigidity of conventional surveys with the responsiveness traditionally associated with qualitative interviewing. In practice, two main formats are emerging: AI-powered surveys and chatbot-driven surveys.

AI-powered surveys integrate natural language processing (NLP) or machine learning into standard survey platforms. The questions are presented on a traditional online survey webpage, but the system understands the nuances of participant responses, identifies relevant keywords or themes, and generates dynamic follow-up questions that delve deeper into specific areas of interest (Chang et al., 2021). These machine learning algorithms can also support researchers in detecting patterns within the data and suggesting question structures to improve quality (Williams, 2024a). A key advantage is the ability to maintain a familiar survey format while allowing for dynamic, tailored probing. This approach is already implemented in platforms such as Voice form.

Chatbot-driven surveys are conducted through a chatbot interface, typically within messaging applications or websites. Instead of a traditional survey link, participants engage in a conversation with a chatbot. These surveys typically involve structured, pre-set questions presented in a conversational format, where the chatbot guides participants through the survey and records responses (Thorne, 2017). (Xiao et al. 2020) argue that it is now commonplace for chatbots to interactively encourage information exchange, and studies show that participants may be more tolerant and patient with an AI companion than with a human researcher (Cui et al., 2017).

While both AI-powered probing and chatbot interfaces represent exciting innovations, they also prompt important theoretical and epistemological questions. In traditional qualitative interviews, probes emerge through researcher reflexivity, empathy, and contextual awareness (Kvale and Brinkmann, 2015). Such interactions rely on rapport, non-verbal cues, and the co-construction of meaning elements AI cannot currently replicate. While AI-generated follow-up questions may be efficient, they are driven by computational logic rather than interpretive judgment and may overlook the affective and relational dimensions of qualitative inquiry.

This is further complicated by the non-deterministic nature of generative chatbots, which can produce different responses and follow-up questions from the same input. This unpredictability raises concerns about reproducibility, as identical prompts may not consistently yield the same outputs, challenging transparency and consistency in qualitative data collection. As (Traum 2017) warns, chatbot misunderstandings could compromise subsequent responses, challenging claims of improved data quality.

Furthermore, the use of AI introduces specific ethical considerations. The data used to train AI models can contain inherent biases, which could be replicated or amplified by the chatbot, potentially leading to a skewed understanding of participants' perspectives, particularly from marginalized groups. There are also unique challenges in gaining informed consent, as researchers must be transparent about who or what participants are interacting with and how their data will be processed. Ultimately, the ethical responsibility remains with the human researcher to not only oversee the AI's output but also to be accountable for any ethical breaches or misinterpretations that arise.

Taken together, these developments point toward a methodological shift in how qualitative researchers might think about survey design. Nonetheless, some scholars argue that AI can serve as a scaffolding tool that extends human capacity rather than replacing it entirely (Zarouali et al., 2024). The potential of AI lies not in its ability to mimic a human researcher, but to assist with tasks that enable the researcher to focus on more complex interpretive work. For instance, an AI could be used to generate a range of potentially probing questions, allowing the researcher to select and refine the most appropriate ones based on their reflexive judgment (Williams, 2024a,b). Similarly, AI could perform initial, high-level coding to identify themes, freeing the researcher to conduct a deeper, more nuanced analysis of context and emotion. This article contributes to the evolving discourse by proposing that AI-driven probing—whether embedded within survey logic or delivered via chatbots—constitutes a hybrid methodological space that remains underexplored. While early findings are promising, more research is needed to explore how participants perceive these tools and how they impact data richness, ethical engagement, and researcher reflexivity in practice (Williams and Ingleby, 2024). These developments reflect a growing shift toward treating AI not just as a technical add-on, but as a methodological actor with implications for how qualitative research is conceptualized, conducted, and interpreted.

## Conclusion

This article has explored the potential of qualitative surveys in social science research and examined how artificial intelligence (AI) can function as a probing tool to enhance the depth and richness of the data collected. Though often overlooked in favor of traditional interviews, qualitative surveys can yield valuable insights into multifaceted social phenomena when designed thoughtfully and ethically. Researchers can address challenges such as survey fatigue and low-quality responses by incorporating AI-powered functionalities such as machine learning algorithms and chatbot-driven surveys, thus improving participant engagement and data quality (Braun et al., 2021).

Online qualitative surveys promote inclusivity and can encourage candid responses, especially on sensitive topics, by providing participants with a sense of anonymity and privacy (Van Deursen and Van Dijk, 2019). However, researchers must consider ethical implications, such as ensuring informed consent and protecting participant confidentiality.

AI shows considerable promise as a probing tool in qualitative surveys, with the potential to generate meaningful dialogue and uncover hidden insights (Zarouali et al., 2024). Whether through AI-powered surveys with follow-up questions based on participant responses or chatbot-driven surveys conducted in a conversational format, these technologies offer innovative ways to engage participants and enhance the research process.

Overall, AI-enhanced qualitative surveys represent a valuable approach in social science research, offering researchers nuanced insights into diverse perspectives and lived experiences (Williams, 2024b). However, continued attention to ethical considerations and methodological rigor is essential to ensure the integrity and validity of research findings in this evolving landscape of data collection methodologies.

This article has argued that AI-powered qualitative surveys, whether through embedded probing tools or conversational chatbots, constitute an underexplored yet increasingly viable method of qualitative data collection. By bridging the scalability of surveys with the dynamic responsiveness of interviews, these approaches challenge the long-standing binary between structured and interpretive methodologies. While these approaches offer clear potential, they also raise important questions about researcher reflexivity, data authenticity, and ethical engagement. As AI continues to evolve, qualitative researchers must remain critically engaged with its use, not only as a technical tool but as a methodological actor with epistemological consequences. Future research should empirically examine participant experiences, response depth, and ethical implications when AI is used to shape the flow and structure of qualitative surveys. Such inquiry is essential for developing robust, transparent, and reflexive practices in an increasingly AI-mediated research landscape.

## Data Availability

The original contributions presented in the study are included in the article/supplementary material, further inquiries can be directed to the corresponding author.
